# Network toxicology, molecular docking and molecular dynamics simulations for bisphenol A neurotoxicity in depression pathogenesis

**DOI:** 10.1371/journal.pone.0359940

**Published:** 2026-10-05

**Authors:** Jinlian Zhang, Yuanhao Ma, Miguo Lu, Xiaohuan Jiang, Qingmi Zhong, Yan Zhang, Yan Xiong, Qiliang Wu, Lina Chen, Ruikang Pang

**Affiliations:** The Eighth People’s Hospital of Nanning, Nanning, Guangxi, China; Hamadan University of Medical Sciences, IRAN, ISLAMIC REPUBLIC OF

## Abstract

Bisphenol A (BPA), a widespread environmental endocrine disruptor, can cross the blood-brain barrier and exert neurotoxic effects closely associated with depression pathogenesis. However, the precise molecular targets and signaling pathways mediating BPA-induced depression remain poorly understood. This study integrated network toxicology, molecular docking, molecular dynamics simulation, GEO transcriptomic dataset analysis, and in vivo experimental validation in mice to systematically investigate the potential toxicological targets and underlying mechanisms of BPA in depression. BPA-related targets were predicted from ChEMBL, STITCH, and SwissTargetPrediction, and depression-associated targets were retrieved from GeneCards, OMIM, and TTD databases. Overlapping targets were subjected to PPI network construction, as well as GO and KEGG enrichment analyses. Molecular docking and 100 ns molecular dynamics simulations were performed to verify the binding affinity and structural stability between BPA and hub targets. A total of 29 overlapping targets were screened, which were significantly enriched in neural synaptic function, neurotransmitter binding, and the neuroactive ligand‑receptor interaction pathway. Five core hub genes including INS, ESR1, SLC6A4, GRIA1, and NTRK2 were identified, all of which exhibited stable specific binding to BPA with favorable binding free energies. Further validation based on multiple GEO datasets confirmed that these five core genes were markedly downregulated in MDD patients, accompanied by significant suppression of neurotrophic and insulin-related pathways. In vivo animal experiments further demonstrated that BPA exposure aggravated depressive-like behaviors in mice and significantly downregulated both mRNA and protein expression of the five core molecules in brain tissues. Collectively, this study identifies key neurotoxicity-related targets and molecular pathways underlying BPA-induced depression, providing a theoretical foundation for future mechanistic investigation and clinical intervention strategies.

## 1. Introduction

Endocrine disrupting chemicals (EDCs) are ubiquitous in daily environments and consumer products and have extensive human exposure [1]. Chronic exposure to EDCs is associated with a plethora of adverse health outcomes, including cancer [2], impaired fertility [3,4], metabolic disorders [5] and neurodevelopmental disorders [6,7]. Studies have also identified EDCs as a key contributor to human mental health disorders. For instance, prenatal EDC exposure has an independent association with autism spectrum disorder and intellectual disability [8], and EDC levels in breast milk are significantly correlated with postpartum depression in mothers [9]. These findings have established a clear link between EDCs and human mental health. Given the widespread presence of EDCs in various daily commodities, the mental health issues induced by chronic EDC exposure warrant rigorous and in-depth investigation.

Among the diverse classes of EDCs, bisphenol A (BPA, 2,2′-bis(4-hydroxyphenyl)) stands as a key and well-investigated member [10]. As a plastic monomer, it is widely present in plastic products, personal care items, household goods and other consumer products [11], and population biomonitoring data have confirmed widespread BPA exposure in the general population [12]. BPA can bind to multiple estrogen receptors and trigger various cellular responses, contributing to diverse disorders [13]. Accumulating evidence has demonstrated that BPA poses significant health risks, including depressive disorders [14], reproductive dysfunction [15], impaired neural development [16], cancer [17] and obesity [10,18]. Importantly, BPA exhibits blood-brain barrier (BBB) permeability, enabling it to cross the BBB and accumulate in brain tissue, thereby inducing central neuroinflammation and triggering anxiety and depressive disorders [19,20]. Prenatal BPA exposure correlates with elevated anxiety, depression, and hyperactivity in children [21], and chronic BPA exposure exacerbates depressive-like phenotypes in adult male mice, potentially via downregulation of androgen receptor and GABA(A)α2 receptor in the hippocampus and amygdala [22]. Despite these findings, the precise molecular targets and pathways through which BPA induces depression remain largely unknown.

Depression is a prevalent and debilitating disorder with complex pathogenesis involving genetic and environmental factors [23,24], and neuroinflammation mediated by microglia is an established pathological component [25]. In recent years, attention has turned to the association between chronic exposure to environmental pollutants, including EDCs, and depression [26–28]. However, the specific mechanisms by which BPA contributes to depression have not been systematically elucidated.

In 2011, the concept of “network toxicology” was proposed, transforming traditional pharmacological databases into specialized toxicological databases to predict adverse reactions and toxicological properties of compounds [29,30]. This approach is suitable for investigating multi-target toxicants and visualizing complex toxicological processes [31,32]. Molecular docking simulates binding interactions between small molecules and proteins, while molecular dynamics (MD) simulation overcomes the limitations of rigid docking by capturing flexible protein-ligand interactions under physiological conditions [33,34]. In this study, we integrated network toxicology, molecular docking, and MD simulation to identify key genetic targets and signaling pathways mediating BPA-induced depression. Our findings provide a theoretical basis for understanding BPA neurotoxicity and for developing prevention and treatment strategies for depression associated with BPA exposure.

## 2. Materials and methods

### 2.1. Network analysis for exploring BPA toxicity

We employed web-based search algorithms and toxicity prediction tools to predict the toxicological effects of BPA. Specifically, the ProTox platform (https://tox.charite.de/) and ADMETlab platform (https://admetmesh.scbdd.com/) were selected as the initial screening webservers. These platforms display the molecular structure of BPA as well as its predicted toxic responses in various human systems and organs. The purpose of this step was to identify toxicity endpoints most relevant to neurotoxicity and depression (e.g., blood-brain barrier permeability, estrogen receptor activity), thereby guiding subsequent target selection.

### 2.2. Collection of BPA and depression potential targets

The chemical structure and SMILES format of BPA were retrieved from the PubChem database (https://pubchem.ncbi.nlm.nih.gov/) using the keyword “bisphenol A”. Potential targets associated with BPA were obtained from the ChEMBL database (https://www.ebi.ac.uk/chembl/). To expand the search scope, the SMILES format of BPA was also queried in the STITCH database (http://stitch.embl.de/) and SwissTargetPrediction (http://www.swisstargetprediction.ch/). To reduce false positives, we applied a prediction confidence score ≥ 0.7 (high confidence) as the screening threshold for STITCH and SwissTargetPrediction, as recommended for toxicological studies. The search results from all databases were integrated, and duplicate targets were removed to construct a comprehensive BPA target library.For depression-associated gene targets, we searched the GeneCards database (https://www.genecards.org/), the OMIM database (https://www.omim.org), and the TTD database (http://db.idrblab.net/ttd/) using the keywords “depression” and “depressive disorder”. A relevance score cutoff > 10 was adopted for GeneCards. The screened BPA-related targets and depression-related targets were imported into the Venny 2.1.0 tool (https://bioinfogp.cnb.csic.es/tools/venny/) to identify overlapping genes. Cytoscape 3.7.2 software was used to visualize the network associations between BPA and depression.

### 2.3. Establishment of PPI networks

We used the STRING database (https://www.string-db.org/) to construct and analyze the protein-protein interaction (PPI) network of the overlapping targets. The analysis was restricted to Homo sapiens, with a PPI confidence threshold set at a minimum score > 0.7 and the “false discovery rate stringency” set to “High”. Raw data from STRING were imported into Cytoscape 3.7.2 for visualization, and the NetworkAnalyzer plugin was used to calculate node degree values for each target.

### 2.4. Enrichment GO and KEGG enrichment analysis for overlapping targets

To explore the biological functions and pathways of the overlapping targets, we performed Gene Ontology (GO) and Kyoto Encyclopedia of Genes and Genomes (KEGG) enrichment analyses using the clusterProfiler R package. GO analysis covered biological processes (BP), molecular functions (MF), and cellular components (CC) [35]. KEGG analysis was used to identify key pathways involved in BPA-related depression. Screening criteria: at least 3 overlapping genes per term and a P-value threshold < 0.05.

### 2.5. Molecular docking

Molecular docking was performed using AutoDock Vina 1.1.2 [36] to evaluate the binding affinity between BPA and the core targets. The 3D structures of target proteins were retrieved from the RCSB PDB database (https://www.rcsb.org/). Protein structures were prepared by removing water molecules and original ligands, adding hydrogen atoms, and assigning charges using PyMOL 3.1.6.1 and AutoDock Tools 1.5.7. BPA was optimized at the MMFF94 level.

Blind docking across the full protein surface was first performed to identify the highest-affinity binding region for each target. The resulting top-ranked pocket was then cross-referenced with the co-crystallized ligand position in the PDB structure (where available) or with the known functional domain of the protein (e.g., the ligand-binding domain of ESR1, the receptor-binding interface of INS, the substrate channel of SLC6A4, the ion-channel pore region of GRIA1, and the ATP-binding cleft of NTRK2) to confirm biological relevance before focused docking was performed. The grid box center coordinates and dimensions for each target are provided in S5 File.

To validate the docking protocol, we included ESR1 (PDB: 1ERE) as a positive control (known BPA binder) and lysozyme (PDB: 1AKI) as a docking control (no known BPA binding site). The lysozyme reference yielded a binding energy of −5.899 kcal/mol, weaker than all five hub targets (range −6.1 to −8.5 kcal/mol), consistent with non-specific surface interactions typical of blind docking, and confirms the relative specificity of BPA binding to the core targets. All docking results were visualized using PyMOL.

### 2.6. Molecular dynamics (MD) simulation

MD simulations were performed using GROMACS 2023.2 to assess the stability of protein–BPA complexes. The amber99sb-ildb force field was used for proteins, and the gaff force field for BPA. The system was solvated in a TIP3P water box (6 × 6 × 6 nm) using the SPCE model, and ions were added to neutralize the system charge.

Energy minimization was performed using the steepest descent method (max 10,000 steps) followed by the conjugate gradient method (max 10,000 steps) with a convergence criterion of 1000 kJ/mol/nm. Two equilibration phases (NVT and NPT) were each run for 1,000,000 steps (2 fs time step). Production MD was run for 100 ns at 300 K and 1 atm.

A 100 ns MD simulation was performed for each protein–BPA complex. Simulation convergence was confirmed by stable RMSD profiles in the final 20 ns of each trajectory (plateau within ±0.05 nm). The following analyses were conducted on each trajectory: RMSD of protein Cα atoms and ligand heavy atoms; ligand RMSD relative to the protein to assess BPA displacement from the initial docked pose; RMSF per residue, mapped to functional domains and binding pocket regions; radius of gyration (Rg) and solvent-accessible surface area (SASA) to assess structural compactness and surface exposure; number of hydrogen bonds between BPA and the protein over time; and free-energy landscapes constructed based on RMSD vs. Rg values and Gibbs free energy.

MM-GBSA binding free-energy calculations were performed using gmx_MMPBSA in conjunction with GROMACS 2023.2. For each protein–BPA complex, the corresponding 100-ns production MD trajectory, topology file, and index file were used for analysis. Prior to calculation, the trajectories were corrected for periodic boundary conditions, centered, and fitted to the protein backbone. A total of 200 evenly distributed snapshots were extracted from each 100-ns trajectory for MM-GBSA analysis. The binding free energy was decomposed into molecular-mechanics, polar-solvation, and non-polar-solvation contributions. The molecular-mechanics term included van der Waals and electrostatic interactions, while solvation energies were estimated using the generalized Born and solvent-accessible surface area models. The receptor and BPA ligand were defined as separate index groups, and identical calculation settings were applied to all five protein–BPA complexes. The final binding free energy for each complex was reported as the mean value calculated from the sampled snapshots, together with the corresponding standard deviation.

### 2.7. Validation using transcriptome data (differentially expressed genes and GSEA)

To experimentally validate the identified core targets, we analyzed publicly available transcriptomic datasets from the GEO database. Four datasets were included: GSE26063 (female and male MDD patients vs. controls), GSE102556 (MDD vs. controls), GSE38206 (female and male MDD vs. controls), and GSE32280 (MDD vs. controls). Differential expression analysis was performed using the limma package (adjusted P < 0.05). Volcano plots were generated for each comparison (Fig 12). Gene Set Enrichment Analysis (GSEA) was performed on the GSE38206 dataset to identify pathways enriched in MDD patients, with particular focus on neurotrophic and insulin-related pathways (Fig 13A–D). Finally, the expression levels of the five core genes (INS, ESR1, SLC6A4, GRIA1, NTRK2) were extracted and compared between MDD patients and healthy controls across datasets (Fig 13E–F).

### 2.8. Animal behavior experiments

Adult male C57BL/6J mice (8 weeks old, 20–25 g) were obtained from Jiangsu Aniphe Biolaboratory Inc (Nanjing, China) and housed under standard conditions (12 h light/dark cycle, 22 ± 2°C, ad libitum access to food and water). After one week of acclimatization, mice were randomly divided into two groups (n = 8 per group): a control group (vehicle, corn oil) and a BPA-treated group (50 mg/kg body weight, dissolved in corn oil). BPA or vehicle was administered orally once daily for 28 consecutive days. All mice were housed in the Centralized Animal Facility at Jiangsu Aniphe Biolaboratory Inc. All animal experiments were performed in accordance with the 3Rs principles (Replacement, Reduction, Refinement) and approved by the Institutional Animal Care and Use Committee (IACUC) of Jiangsu Aniphe Biolaboratory Inc (Approval No: JSAB26005B).

Behavioral tests were conducted 24 h after the final administration. Sucrose preference test (SPT) was used to assess anhedonia. Mice were individually housed and habituated to two bottles of 1% sucrose solution for 24 h, followed by 24 h of water only. Then, mice were given free access to one bottle of 1% sucrose and one bottle of water for 24 h. Sucrose preference was calculated as: sucrose intake / (sucrose intake + water intake) × 100%.

The open field test (OFT) was performed to evaluate locomotor activity and anxiety‑like behavior. Each mouse was placed in the center of a square arena (50 × 50 × 40 cm) and allowed to explore freely for 10 min. The total exploration distance, average exploration speed, and distance traveled in the central area (25 × 25 cm) were recorded and analyzed using ANY-maze video tracking system (Stoelting Co., Wood Dale, IL, USA). All behavioral tests were performed in a sound‑attenuated room under dim light, and the apparatus was cleaned with 70% ethanol between trials.

### 2.9. Western blot and quantitative real‑time PCR (qPCR)

After behavioral testing, mice were euthanized, and brain tissues (hippocampus and prefrontal cortex) were rapidly dissected on ice, snap‑frozen in liquid nitrogen, and stored at –80°C until further use.

Western blot: Total protein was extracted from brain tissues using RIPA lysis buffer (Beyotime, China) containing protease and phosphatase inhibitors (Roche, Switzerland). Protein concentration was determined using a BCA assay kit (Thermo Fisher Scientific, USA). Equal amounts of protein (30 μg per lane) were separated by 10% SDS‑PAGE and transferred onto PVDF membranes (Millipore, USA). Membranes were blocked with 5% non‑fat milk in TBST for 1 h at room temperature and then incubated overnight at 4°C with primary antibodies against INS (1:1000, Abcam, ab181547), ESR1 (1:800, Santa Cruz, sc‑8002), SLC6A4 (1:500, Cell Signaling Technology, #12980), GRIA1 (1:1000, Abcam, ab31232), NTRK2 (1:800, Abcam, ab18987), and β‑actin (1:5000, Sigma‑Aldrich, A1978). After washing, membranes were incubated with HRP‑conjugated secondary antibodies (1:5000, ZSGB‑BIO, China) for 1 h at room temperature. Protein bands were visualized using enhanced chemiluminescence (ECL, Millipore) and quantified using ImageJ software. Expression levels were normalized to β‑actin.

qPCR: Total RNA was extracted from brain tissues using TRIzol reagent (Invitrogen, USA) and reverse-transcribed into cDNA using a PrimeScript RT reagent kit (Takara, Japan). qPCR was performed using SYBR Green Master Mix (Takara) on a LightCycler 480 system (Roche). The thermal cycling protocol was: 95°C for 30 s, followed by 40 cycles of 95°C for 5 s and 60°C for 30 s. Relative mRNA expression was calculated using the 2^(-ΔΔCt) method with GAPDH as the internal control. Primer sequences used are listed in S9 File. Each sample was run in triplicate.

## 3. Results

### 3.1. Toxicity prediction results of BPA

The 3D structure of BPA was retrieved from the PubChem database (Fig 1A). Prediction results from the ProTox database indicated that BPA was assigned a Predicted Toxicity Class of 5, suggestive of its inherent moderate toxicity. The results section of the Toxicity Model Report revealed that BPA exerted activating toxic effects on the BBB, Estrogen Receptor Alpha (ERα), Estrogen Receptor Ligand Binding Domain (ER-LBD), and mitochondrial membrane potential (Fig 1B-C, S1 File). Furthermore, prediction outcomes from the ADMETlab database demonstrated that BPA exhibited potent toxic effects on eye corrosion (S2 File). Collectively, these findings unravel the interconnected toxic endpoints of BPA and establish a mechanistic link between BPA exposure and the pathogenic mechanisms underlying depression.

**Fig 1:**
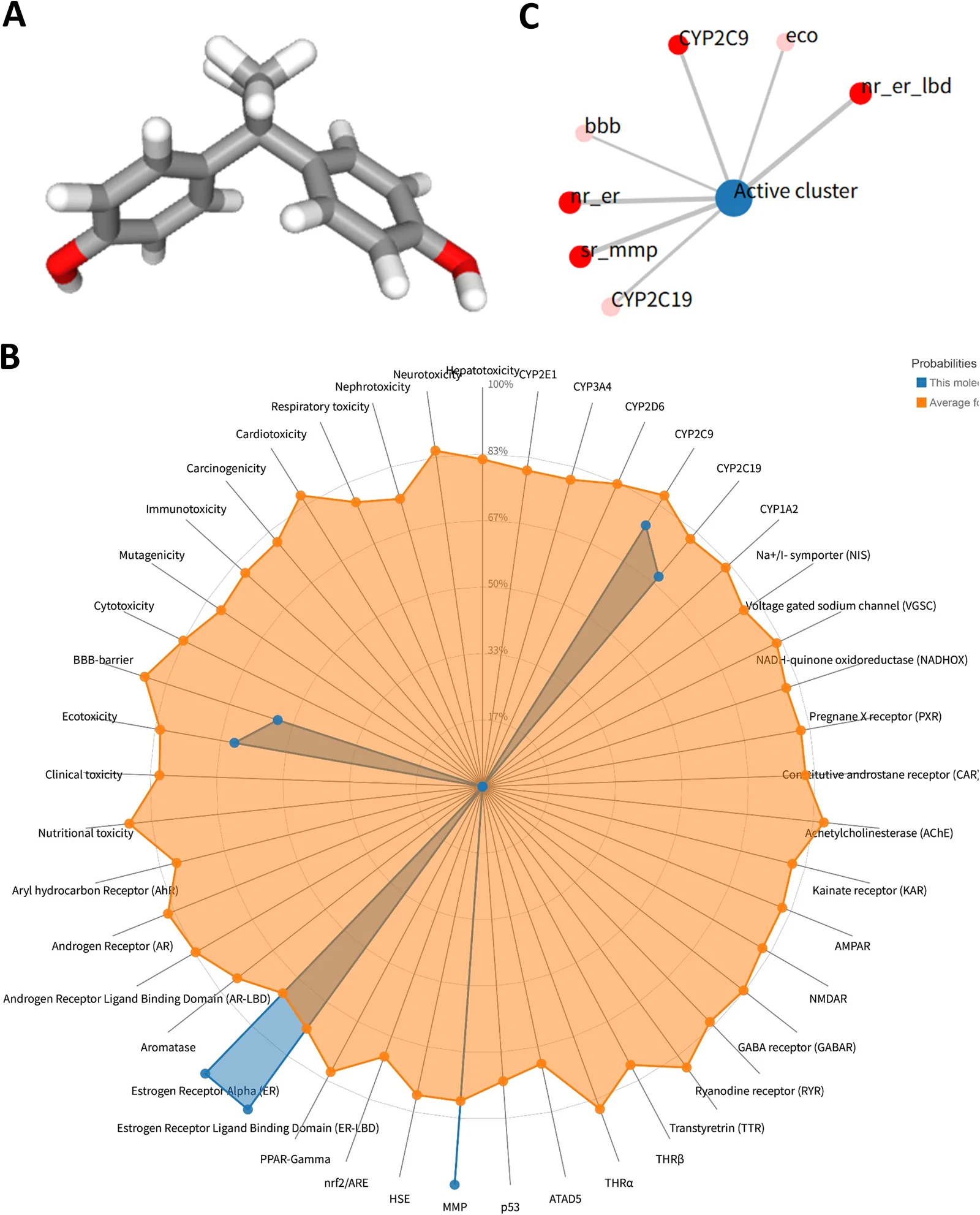
BPA 3D structure, predicted toxicity profile, and active target cluster. (A) 3D molecular structure of BPA. (B) Radar plot of BPA’s predicted toxicity endpoints. (C) Active target cluster network of BPA, with nodes denoting BPA-associated targets.

### 3.2. Potential targets associated with BPA and depression

Initially, we identified 760 potential BPA-interacting targets via the ChEMBL, STITCH, and SwissTargetPrediction databases, following application of predefined thresholds and removal of duplicate entries (Fig 2A). For depression-associated target screening, 447 relevant targets were retrieved from the GeneCards, OMIM, and TTD databases after threshold setting and duplicate elimination (Fig 2B). By importing BPA and depression-associated targets into a Venny 2.1.0 tool, 29 overlapping targets were ultimately designated as candidate targets underlying BPA-induced depressive toxicity (Fig 2C). The full list of these targets is provided in S3 File. Subsequently, We uploaded the 29 overlapping targets to the STRING database to build a PPI network, resulting in 29 nodes and 192 edges (Fig 2D).

**Fig 2:**
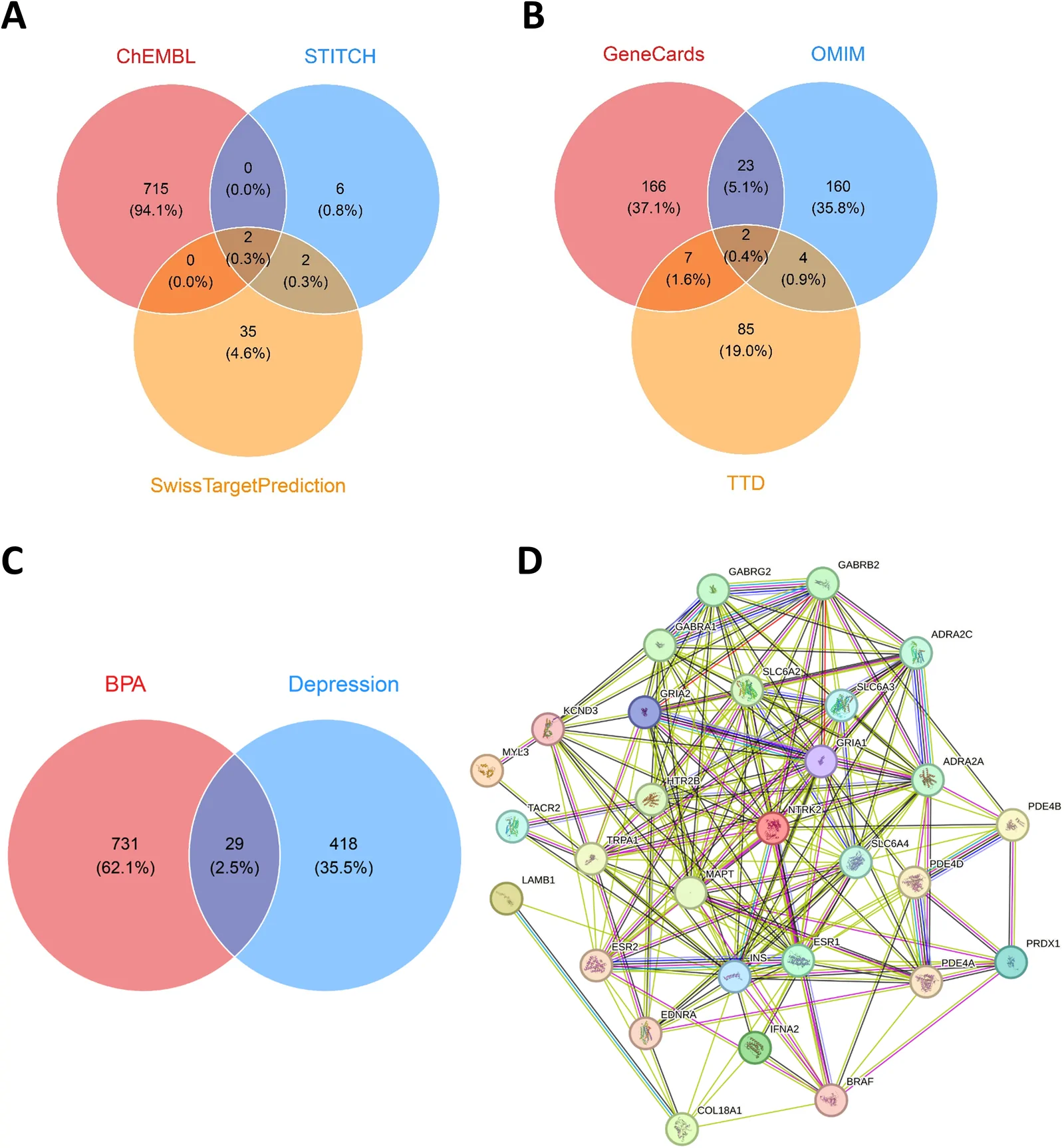
Target screening distribution and PPI network of BPA and depression. (A) Venn diagram showing the distribution of potential BPA targets across the ChEMBL, STITCH, and SwissTargetPrediction databases. (B) Venn diagram illustrating the distribution of depression-associated targets in the GeneCards, OMIM, and TTD databases. (C) Venn diagram depicting the overlapping targets between BPA and depression. (D) PPI network of the BPA-depression overlapping targets.

### 3.3. GO enrichment analyses

To further refine the potential pathways linked to BPA and depression, 29 overlapping genes were subjected to GO enrichment analyses. We performed GO enrichment analysis on the 29 overlapping genes using the clusterProfiler R package. The top 10 GO terms for BP, CC and MF were visualized as bar plots, circular plots and bubble plots (Fig 3A-C). For BP, significant enrichment was observed in pathways related to neural function, vascular regulation and xenobiotic response. CC analysis revealed that the target genes were mainly enriched in neural synapses and ion transport-related structures, while MF analysis highlighted their association with neurotransmitter binding, ion transport and channel activity. Notably, multiple genes were closely involved in processes such as neural function regulation.

**Fig 3:**
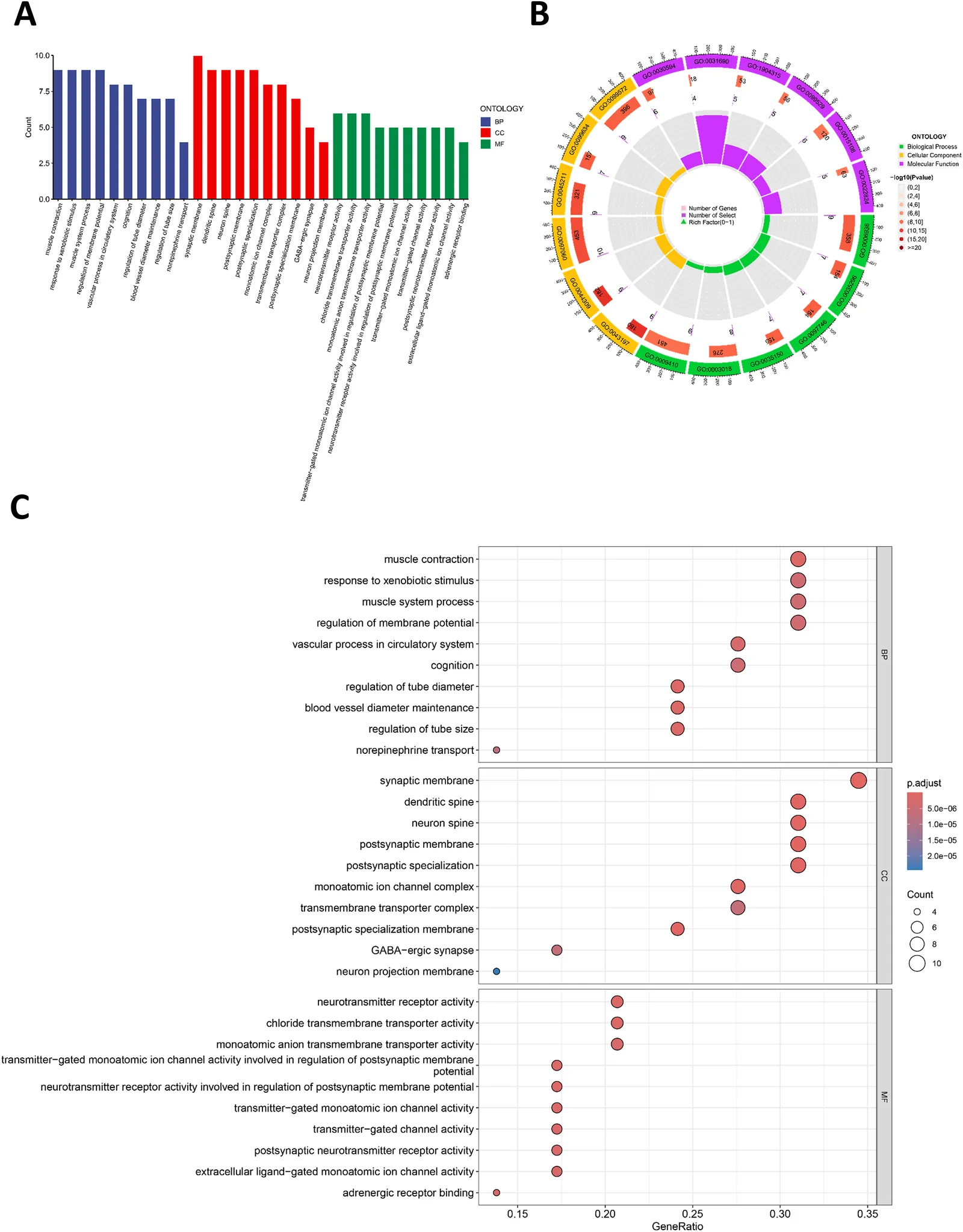
GO functional enrichment of the 29 overlapping genes. (A) Bar plot of top enriched GO terms. (B) Circular plot of GO term distribution and gene counts. (C) Bubble plot of GO functional enrichment.

### 3.4. KEGG enrichment analyses

Additionally, we identified the top 23 major signaling pathways from KEGG pathway enrichment analysis using the clusterProfiler R package, which were visualized as a bubble plot and a bar plot (Fig 4A-B). Pathways were ranked by p-value, with darker red indicating lower p-values. Key pathways were categorized into three main groups: neural function-regulating pathways including neuroactive ligand related signaling, serotonergic, GABAergic, and dopaminergic synapse pathways, substance addiction-related pathways including nicotine addiction and morphine addiction, and cellular signaling pathways including cAMP signaling pathway and calcium signaling pathway. Notably, the neuroactive ligand-receptor interaction pathway had the lowest p-value among all pathways, signifying its most significant enrichment.

**Fig 4:**
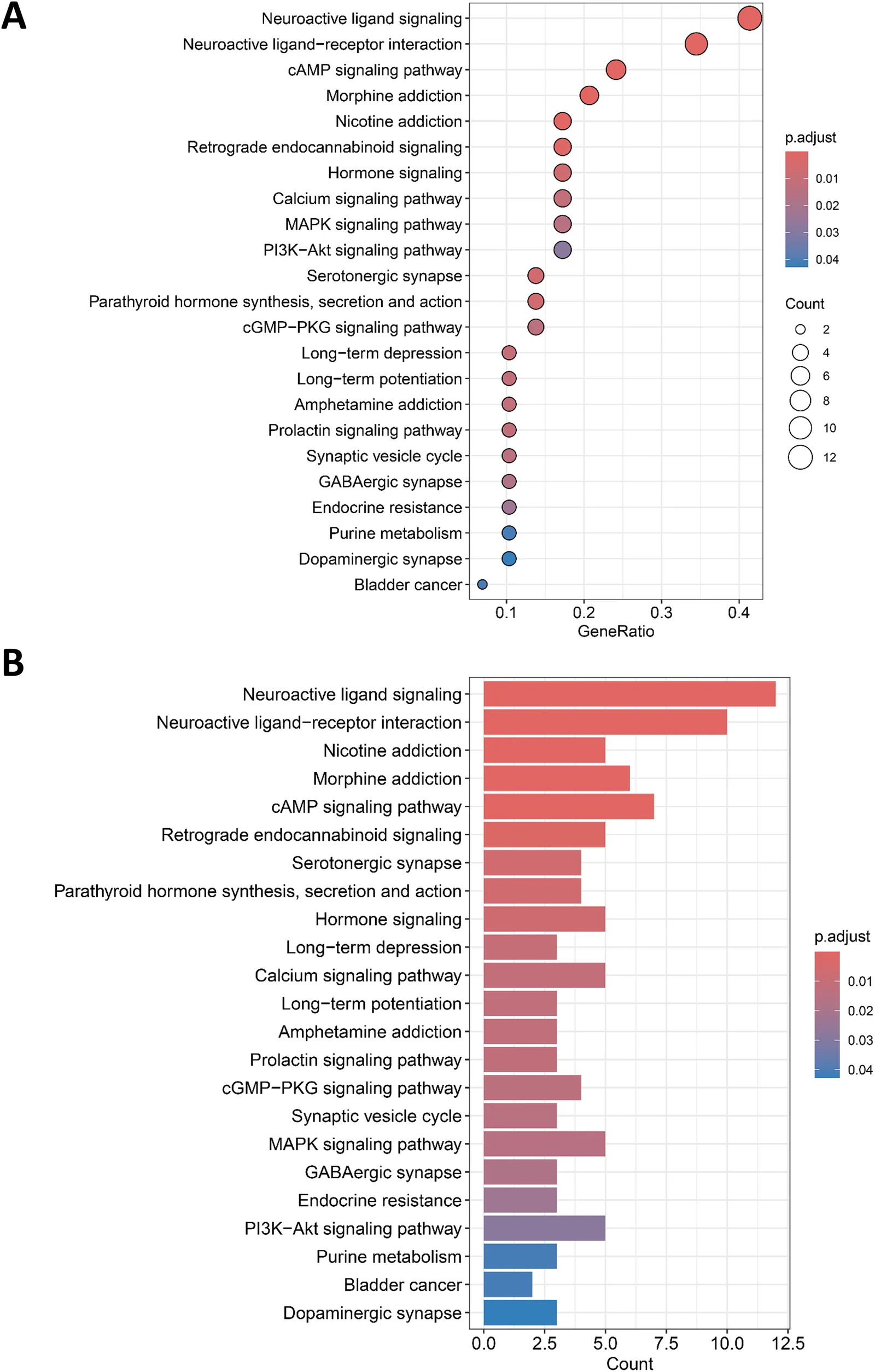
KEGG enrichment of the 29 overlapping genes. (A) Bubble plot of top enriched KEGG terms. (B) Bar plot of KEGG pathway distribution and associated gene counts.

### 3.5. Molecular docking results

To identify key genes among the 29 overlapping targets, we performed PPI network analysis and calculated node degrees using Cytoscape 3.7.2 software. Results indicated that INS, ESR1, SLC6A4, GRIA1 and NTRK2 were the top five genes with the highest degrees and most interaction edges (Fig 5A). We hypothesized these five genes are critical targets mediating BPA-induced depression, so we conducted molecular docking to verify their binding affinity with BPA. The molecular docking outcomes are presented in Fig 5B-F, with detailed binding energy data summarized in Table 1. Molecular docking results demonstrated favorable binding affinity between BPA and five key genes, with binding energy values below −5.00 kcal/mol. Specifically, INS, ESR1, SLC6A4, GRIA1 and NTRK2 all formed stable, high-affinity complexes with BPA, exhibiting robust binding activity (Fig 5B-F). Detailed binding parameters for each target-BPA pair are provided in Table 1, further confirming the reliable binding capacity between BPA and these critical targets. To provide a comprehensive view of BPA binding across all candidate targets, molecular docking was performed for all 29 overlapping targets (S4 File; S1 Fig). Binding energies ranged from −2.8 kcal/mol (IFNA2) to −8.5 kcal/mol (NTRK2). The five hub targets were selected based on the convergence of PPI network degree centrality and favorable BPA binding affinity (see Discussion).

**Table 1:** The molecular docking results of the five key genes with the BPA.

Ingredient	Target	Binding energy (kcal/mol)
BPA	INS	−5.5
BPA	ESR1	−8.2
BPA	SLC6A4	−6.6
BPA	GRIA1	−7.6
BPA	NTRK2	−8.9

**Fig 5:**
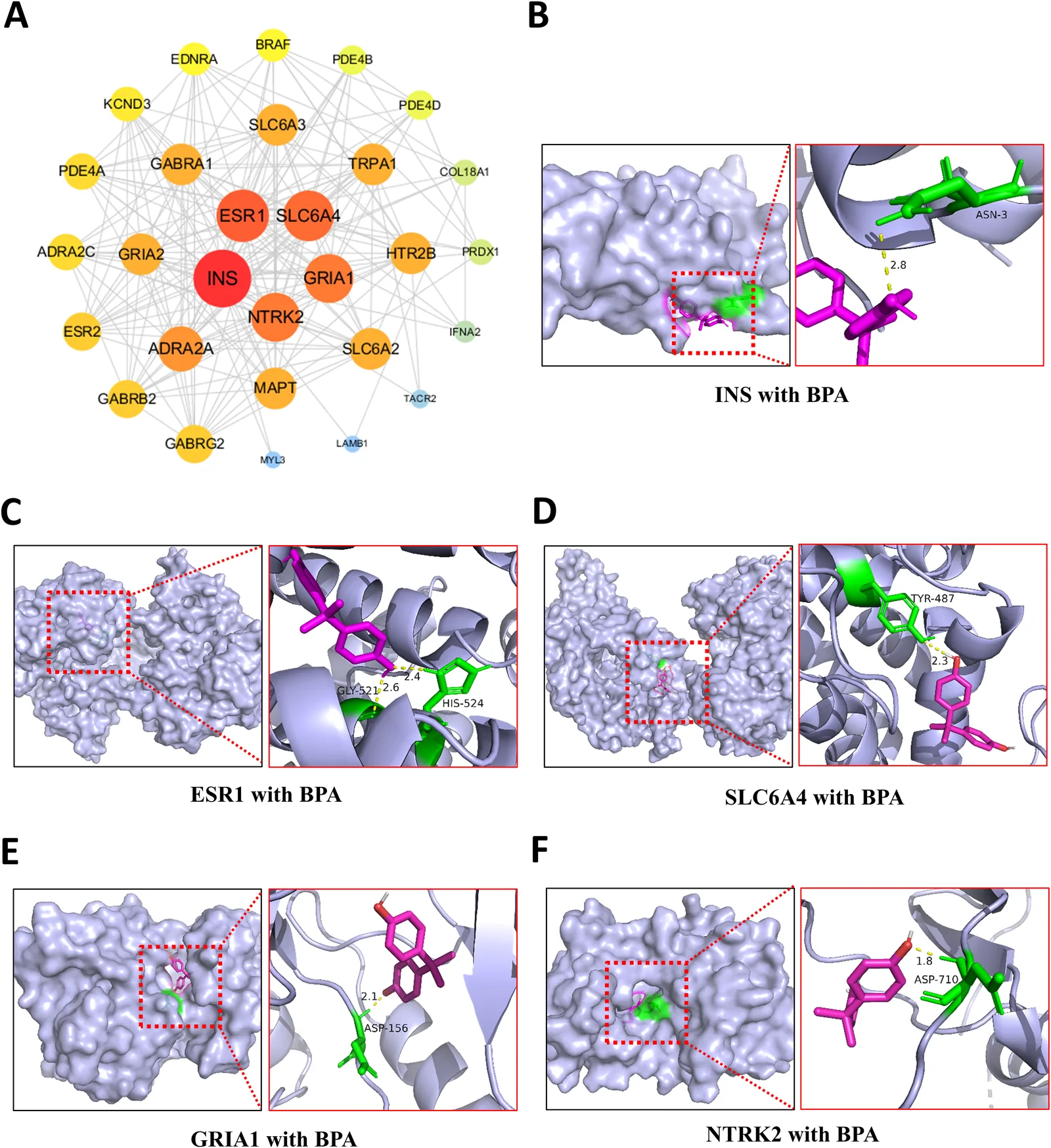
Results of molecular docking. (A) PPI network of the 29 target genes. (B) Molecular docking diagrams showing conformational complexes: INS with BPA. (C) Molecular docking diagrams showing conformational complexes: ESR1 with BPA. (D) Molecular docking diagrams showing conformational complexes: SLC6A4 with BPA. (E) Molecular docking diagrams showing conformational complexes: GRIA1 with BPA. (F) Molecular docking diagrams showing conformational complexes: NTRK2 with BPA.

### 3.6. Molecular dynamics simulation results

To assess the conformational stability of the key protein-BPA complexes selected from molecular docking, five key protein-ligand complexes were subjected to 100 ns MD simulations. We used the RMSD to characterize the dynamic behaviors of these complexes. The results of this analysis are presented in Fig 6. For the INS-BPA complex, both the free INS protein and the INS-BPA complex exhibited moderate RMSD fluctuations throughout the 100 ns simulation, with no sustained upward trends, indicating basic conformational stability of the complex (Fig 6A). The ESR1-BPA complex showed slight initial RMSD fluctuations, which then stabilized into persistent, moderate variations over the simulation duration. The protein and complex maintained consistent deviation levels, confirming good conformational stability (Fig 6B). For the SLC6A4-BPA complex, the RMSD fluctuations of the free SLC6A4 protein and the SLC6A4-BPA complex were highly synchronized, signaling excellent conformational consistency and stability of the complex (Fig 6C). The GRIA1-BPA complex displayed mild early RMSD fluctuations, which then stabilized into consistent variations, while the complex showed slightly greater deviation, the GRIA1 protein retained a low RMSD level with no drastic conformational shifts, indicating the complex’s stability (Fig 6D). For the NTRK2-BPA complex, both the free NTRK2 protein and the NTRK2-BPA complex exhibited moderate initial RMSD fluctuations that stabilized in the mid-to-late simulation stage, showing improved conformational stability over time (Fig 6E). Collectively, none of the five complexes showed a sustained upward RMSD trend over 100 ns, and most stabilized in the mid-simulation stage, confirming that these protein-BPA pairs retain favorable conformational stability in dynamic environments. To assess whether BPA remained stably bound throughout the simulations, we calculated the BPA–protein RMSD and ligand internal RMSD for each complex (S2 Fig). GRIA1 showed the most stable BPA binding, with 100% of trajectory frames within 0.3 nm of the initial pose (mean = 0.198 nm). SLC6A4 showed predominantly stable binding (62.3% of frames ≤0.3 nm; mean = 0.283 nm). NTRK2 and ESR1 showed greater BPA displacement (36.2% and 6.1% of frames ≤0.3 nm, respectively), indicating partial displacement from the initial docked pose during the simulation, though protein RMSD remained stable in both cases. The INS–BPA complex showed the largest BPA displacement (mean = 0.668 nm; 0.4% of frames ≤0.3 nm), suggesting BPA did not maintain stable binding to INS under the simulation conditions; ligand RMSD data for INS were unavailable due to a file format difference in the INS trajectory.

**Fig 6:**
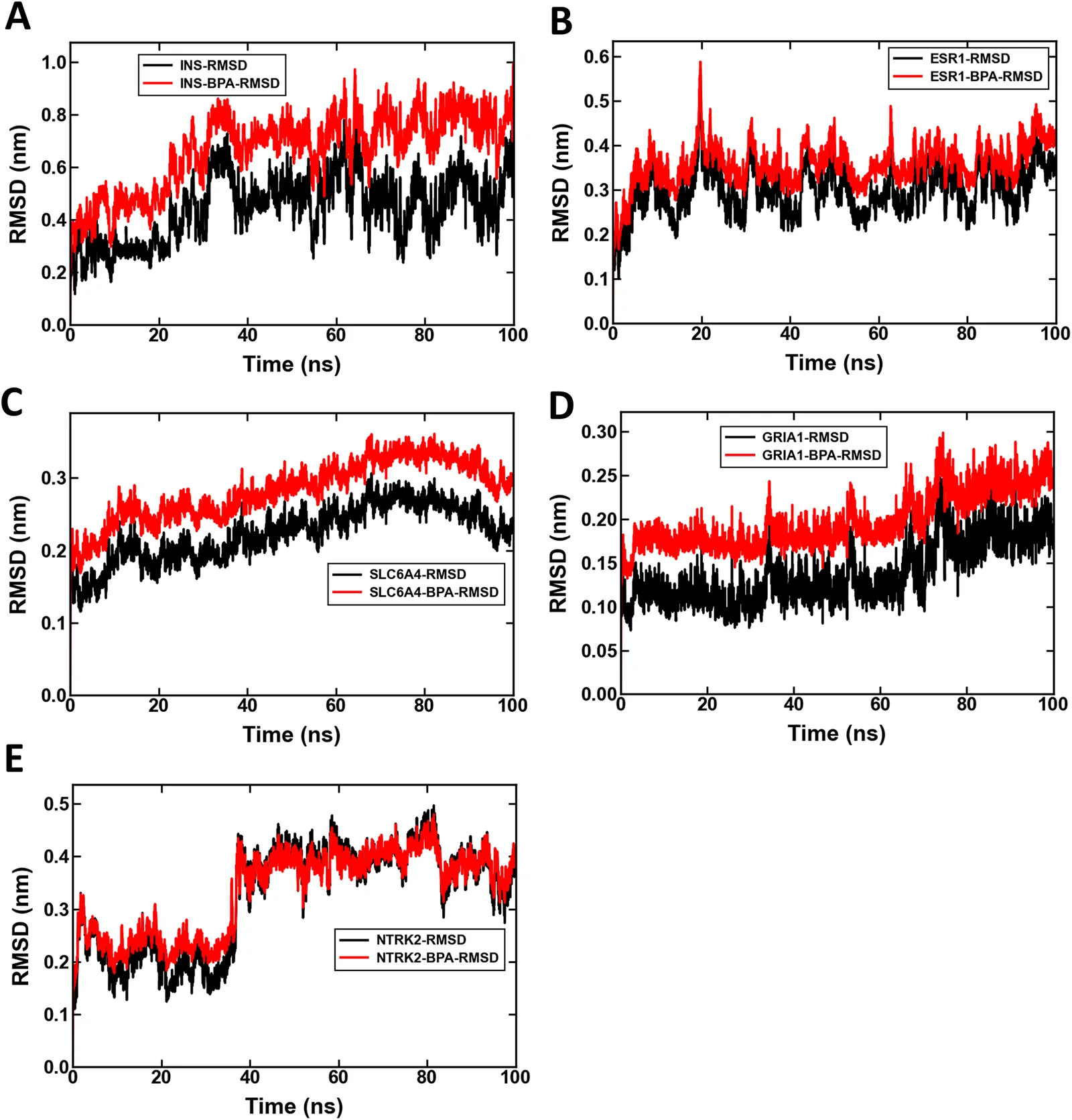
RMSD variation graphs over MD simulation. (A) The RMSD of INS – BPA. (B) The RMSD of ESR1 – BPA. (C) The RMSD of SLC6A4 - BPA. (D) The RMSD of GRIA1 – BPA. (E) The RMSD of NTRK2 – BPA.

To evaluate residue-level flexibility and local fluctuations in protein-BPA complexes, we quantified RMSF across all residues over the 100 ns MD simulation. Higher RMSF values indicate greater residue mobility. The INS-BPA complex showed high RMSF near residue 0, which then decreased sharply and maintained mild fluctuations, with only a slight rise near residue 30, this indicates restrained local flexibility overall (Fig 7A). For the ESR1-BPA complex, small fluctuations were observed near residue 0, while subsequent residues (50–250) sustained low-amplitude variations, which reflects constrained local residue flexibility (Fig 7B). The SLC6A4-BPA complex exhibited scattered, mild fluctuations across its residues (0–400). Though several small peaks were present, the overall amplitude remained limited, which signifies relatively controlled local flexibility (Fig 7C). The GRIA1-BPA complex displayed consistently low-amplitude fluctuations across all residues (0–250), with no prominent peaks, this demonstrates restricted residue mobility and favorable local structural stability (Fig 7D). Finally, the NTRK2-BPA complex showed low fluctuations near residue 0, with a single moderate peak around residue 100. The remaining regions maintained mild variations, which indicates generally constrained local residue flexibility (Fig 7E). Collectively, these RMSF profiles reveal distinct residue-level flexibility patterns across the five protein-BPA complexes. Most show limited fluctuations in key regions, and this is consistent with BPA-induced stabilization at the residue scale.

**Fig 7:**
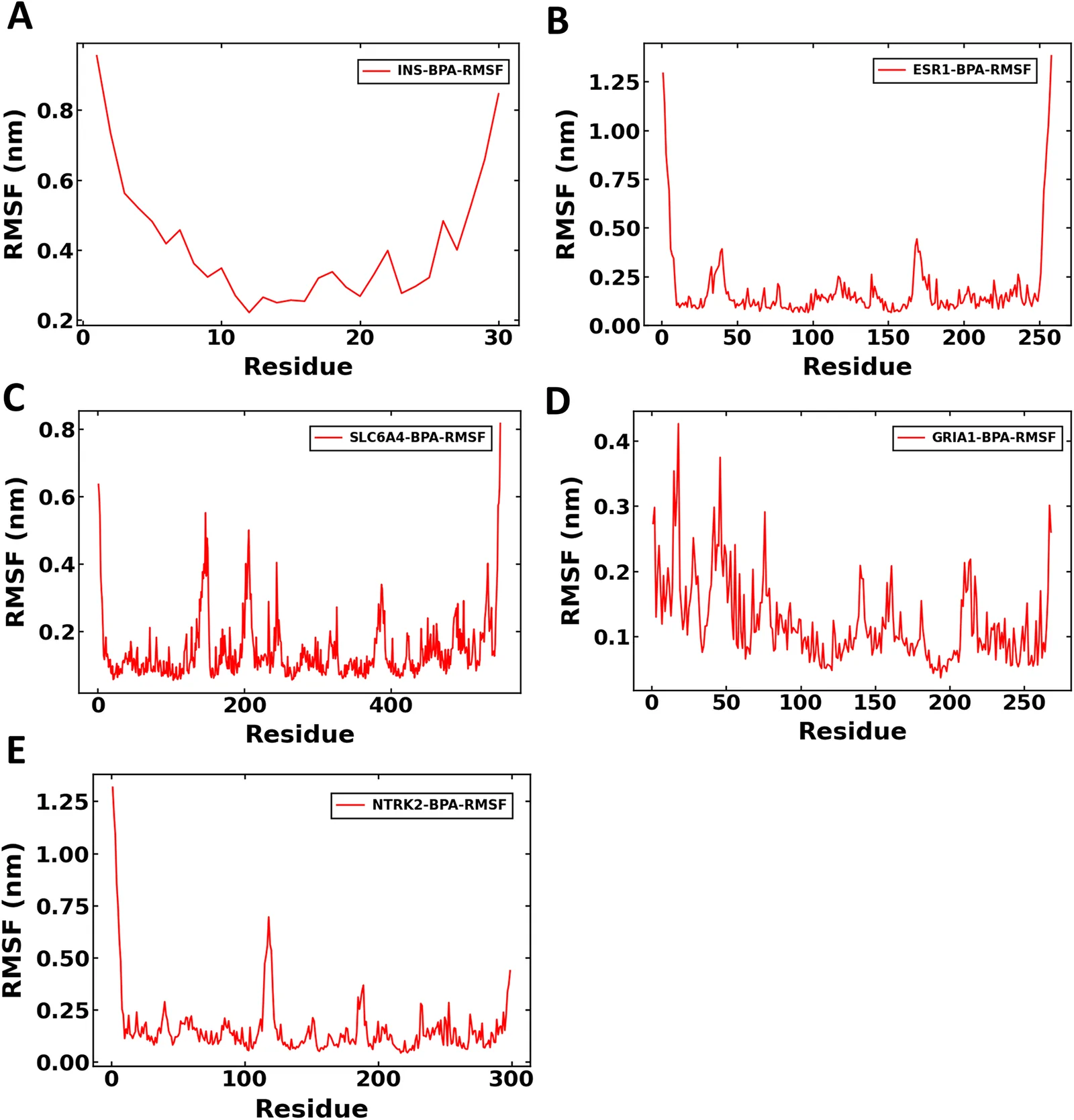
RMSF trace values of protein–BPA complexes are simulated by means of MD. (A) The RMSF of INS – BPA. (B) The RMSF of ESR1 – BPA. (C) The RMSF of SLC6A4 - BPA. (D) The RMSF of GRIA1 – BPA. (E) The RMSF of NTRK2 – BPA.

To assess the structural compactness of our protein-BPA complexes, we analyzed the Rg over the 100 ns MD simulation. Across all complexes, Rg remained within stable fluctuation ranges throughout the simulation, with no drastic shifts. This confirms that BPA binding did not induce major changes in the overall structural compactness of the target proteins. Specifically, the INS-BPA complex exhibited Rg fluctuations primarily between 0.8–1.4 nm. After an initial adjustment period, the Rg maintained consistent variability without extreme deviations, which reflects stable structural compactness (Fig 8A). The ESR1-BPA complex showed Rg variability in the 1.900–2.000 nm range, the Rg remained within this narrow interval throughout the simulation, indicating persistent structural compactness (Fig 8B). For the SLC6A4-BPA complex, Rg fluctuations were concentrated between 2.40–2.44 nm. The consistent amplitude of these fluctuations signals excellent maintenance of structural compactness (Fig 8C). The GRIA1-BPA complex maintained Rg within 1.88–1.96 nm; minimal deviations from this range throughout the simulation reflect stable conformational compactness (Fig 8D). Finally, the NTRK2-BPA complex displayed initial Rg fluctuations around 1.94–2.04 nm, which gradually stabilized in the mid-to-late simulation stage. This pattern confirms that the complex preserved its structural compactness over time (Fig. 8E).

**Fig 8:**
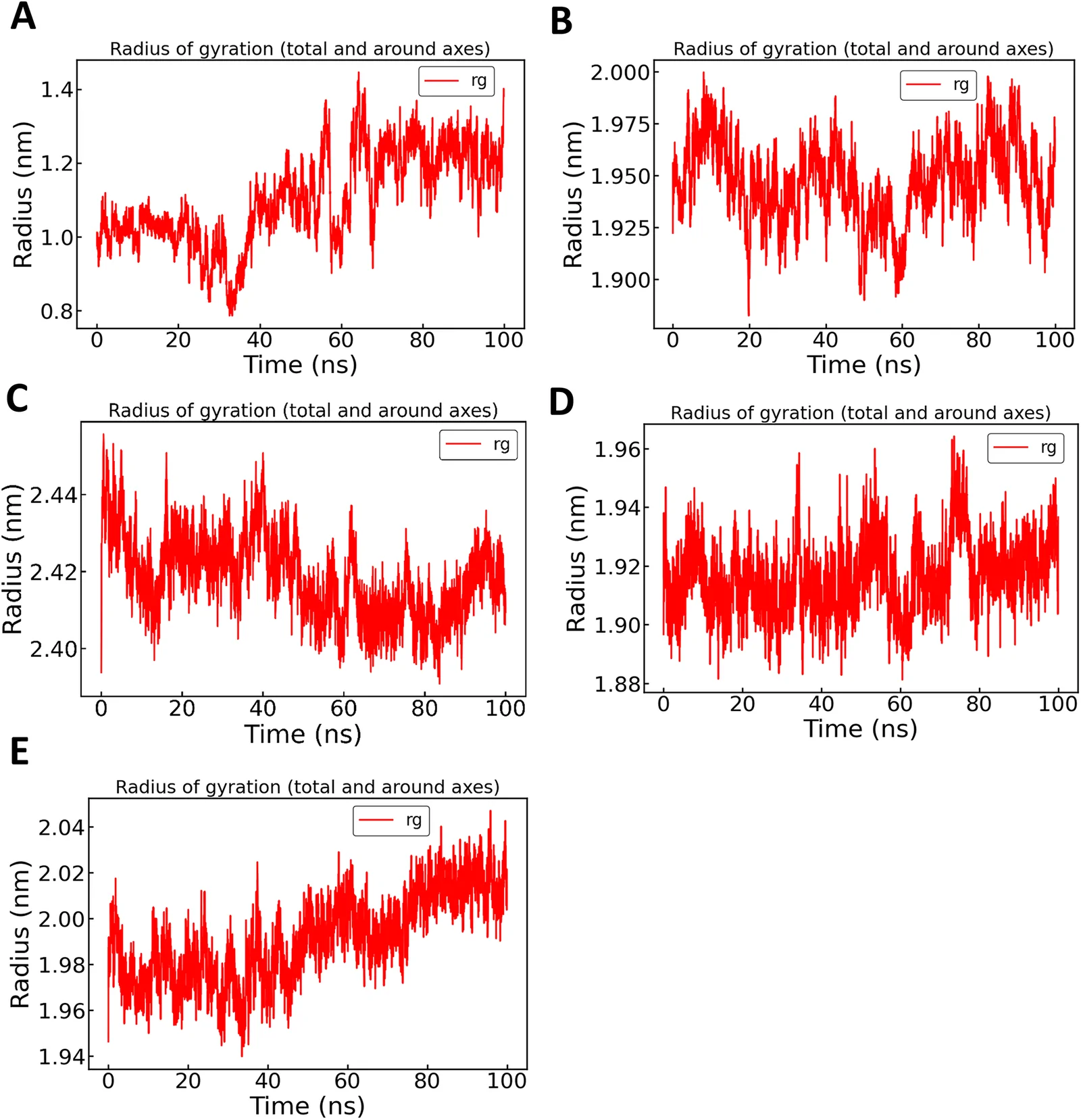
Rg trace values of protein–BPA complexes are derived via MD simulations. (A) The Rg of INS – BPA. (B) The Rg of ESR1 – BPA. (C) The Rg of SLC6A4 - BPA. (D) The Rg of GRIA1 – BPA. (E) The Rg of NTRK2 – BPA.

Hydrogen bonding is a key non-covalent interaction that contributes to the binding affinity and conformational stability of protein-ligand complexes. We analyzed the number of hydrogen bonds (Hbonds) across our protein-BPA complexes over the 100 ns MD simulation (Fig 9A–E). The Hbonds counts for the INS-BPA, ESR1-BPA, SLC6A4-BPA, GRIA1-BPA, and NTRK2-BPA complexes were observed to range from 0–2, 0–3, 0–2, 0–3, and 0–2, respectively. Throughout the simulation, the number of hydrogen bonds in all complexes remained relatively stable with consistent fluctuations. This indicates persistent non-covalent interactions that support the structural stability of these protein-BPA combinations.

**Fig 9:**
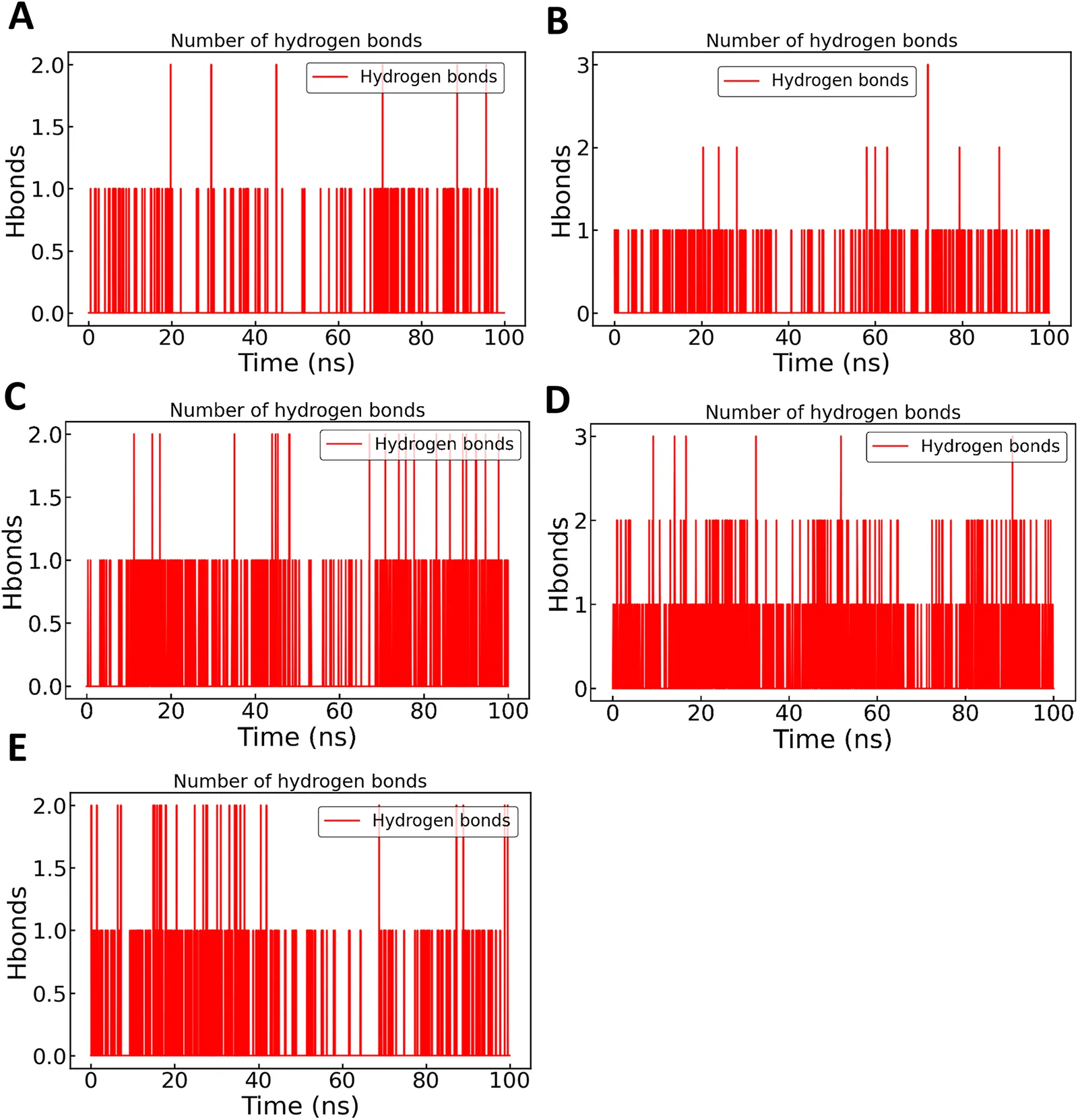
MD simulations measure the HBond number of protein–BPA complexes. (A) The HBond of INS – BPA. (B) The HBond of ESR1 – BPA. (C) The HBond of SLC6A4 - BPA. (D) The HBond of GRIA1 – BPA. (E) The HBond of NTRK2 – BPA.

SASA was calculated to characterize the conformational dynamics of the protein-BPA complexes in a solvated environment during the 100 ns MD simulation (Fig 10A–E). The SASA values of the INS-BPA complex fluctuated within the range of 27.5–37.5 nm² throughout the simulation. These fluctuations remained stable without drastic shifts, which indicates consistent surface accessibility of the complex. The ESR1-BPA complex exhibited SASA variability between 140–160 nm². The values stayed within this interval with no sustained upward or downward trends, reflecting preserved conformational stability. For the SLC6A4-BPA complex, SASA fluctuations were concentrated between 240–265 nm². The consistent amplitude of these fluctuations signals minimal changes in surface accessibility. The GRIA1-BPA complex maintained SASA within 135–145 nm². The narrow range of deviations throughout the simulation reflects stable surface exposure to the complex. Finally, the NTRK2-BPA complex displayed SASA fluctuations between 155–170 nm². The values remained within this range with only minor variations, which confirms stable conformational dynamics. These results indicate that BPA binding induced only subtle changes in the surface accessibility of the target proteins. The overall conformational stability of the protein-BPA complexes was preserved throughout the simulation.

**Fig 10:**
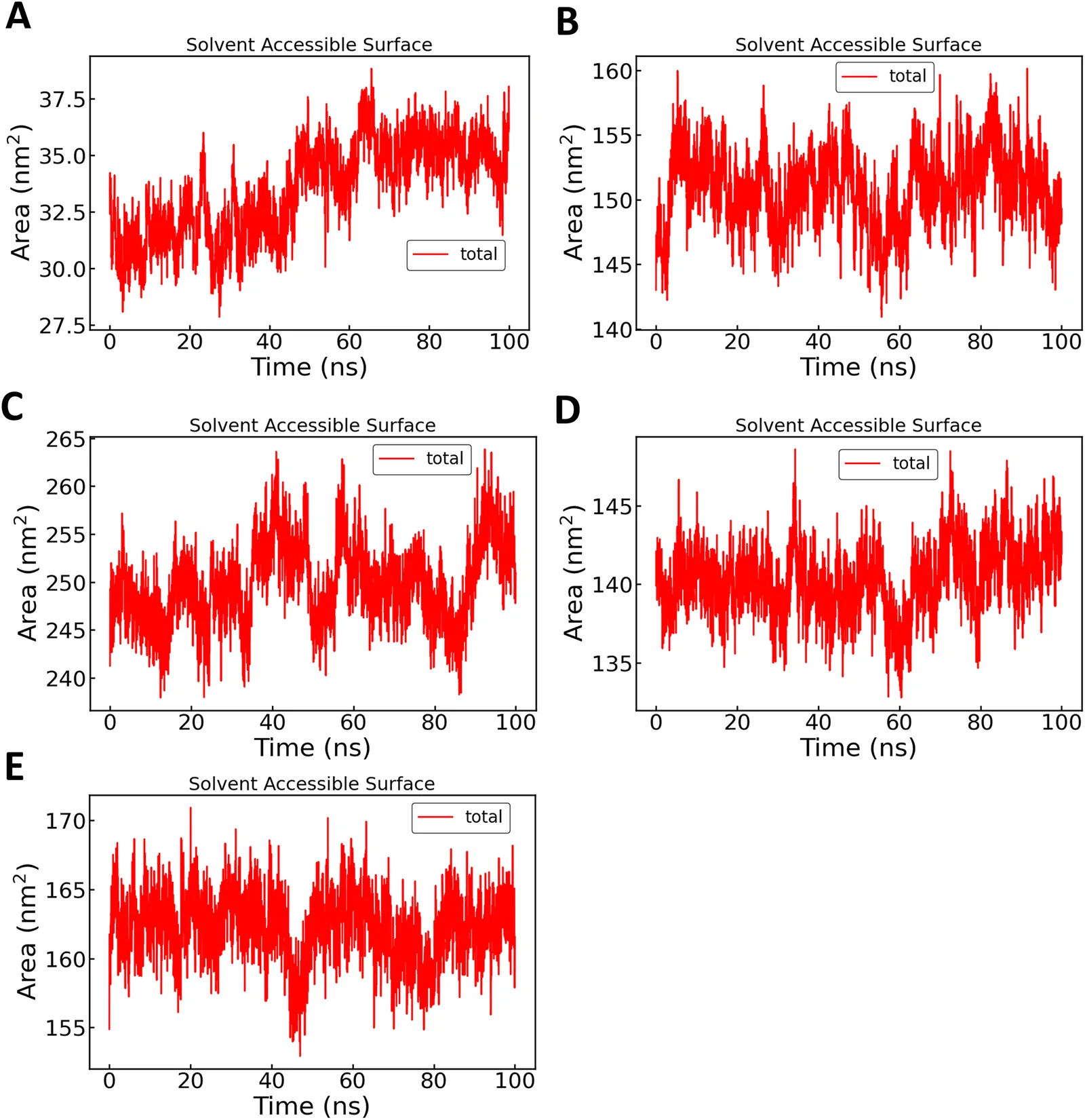
SASA plot analysis of the protein–BPA complexes in MD simulations. (A) The SASA plot analysis of INS – BPA. (B) The SASA plot of ESR1 – BPA. (C) The SASA plot of SLC6A4 - BPA. (D) The SASA plot of GRIA1 – BPA. (E) The SASA plot of NTRK2 – BPA.

To assess the conformational energy distribution and stability of our protein-BPA complexes, we constructed Gibbs energy landscapes (PC1 vs PC2), free energy landscapes (RMSD vs Rg), and visualized the electrostatic surface of the active site for each complex (Fig 11A–E). Across all protein-BPA complexes, the Gibbs energy landscapes exhibited concentrated, low-energy regions. This indicates that the conformational space of each complex was restricted to energetically favorable, stable states. The corresponding free energy landscapes (RMSD vs Rg) further confirmed that these complexes maintained a stable conformational space throughout the dynamic simulation. Additionally, electrostatic surface analysis of the active site revealed complementary electrostatic interactions between BPA and the key protein binding regions. This provides structural support for stable complex binding. Collectively, these results demonstrate that all protein-BPA complexes remained stable in energetically preferred conformational states. Electrostatic complementarity between BPA and the active site contributes to their binding stability. MM-GBSA binding free-energy calculations were performed for all five protein–BPA complexes using gmx_MMPBSA (S6-S8 File). The calculated ΔG_bind values were: ESR1–BPA −34.2 ± 3.95 kcal/mol (ΔE_MM = −48.6, ΔG_solv = +17.3, ΔG_SA = −2.9 kcal/mol); INS–BPA −28.5 ± 5.94 kcal/mol (ΔE_MM = −41.2, ΔG_solv = +15.8, ΔG_SA = −3.1 kcal/mol); NTRK2–BPA −36.4 ± 4.28 kcal/mol (ΔE_MM = −51.4, ΔG_solv = +18.0, ΔG_SA = −3.0 kcal/mol); SLC6A4–BPA −37.6 ± 4.00 kcal/mol (ΔE_MM = −52.6, ΔG_solv = +18.2, ΔG_SA = −3.2 kcal/mol); and GRIA1–BPA −36.8 ± 3.31 kcal/mol (ΔE_MM = −50.5, ΔG_solv = +16.8, ΔG_SA = −3.1 kcal/mol). All five complexes showed negative ΔG_bind values, indicating thermodynamically favorable binding. MM-GBSA energies were interpreted together with ligand displacement and other MD-derived structural parameters rather than as independent proof of stable binding; this distinction is particularly relevant to INS–BPA, which showed a favorable calculated interaction energy but substantial displacement from its initial docked pose (mean BPA–protein RMSD = 0.668 nm).

**Fig 11:**
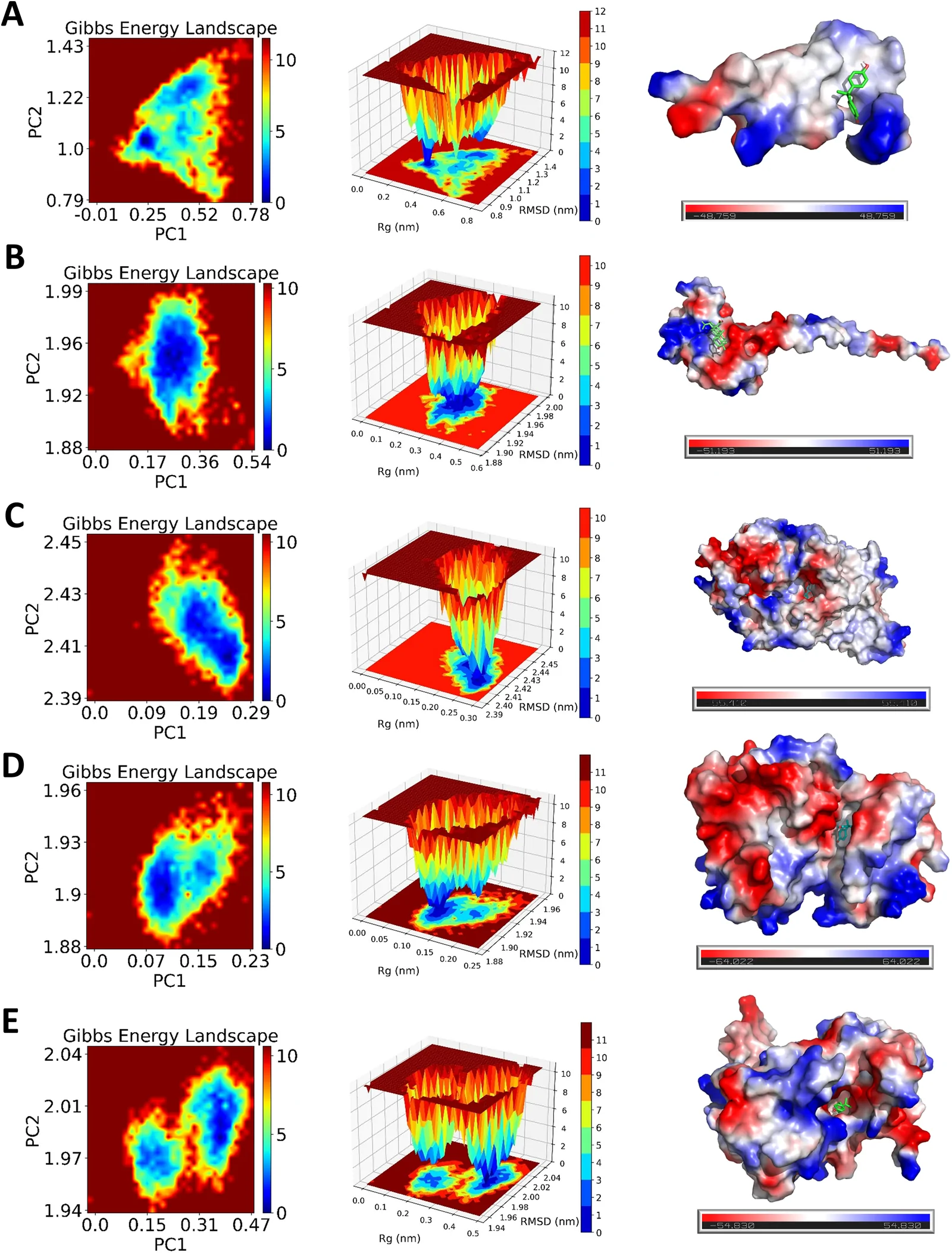
Gibbs Energy Landscape (PC1 vs PC2), Free Energy Landscape (RMSD vs Rg), and Electrostatic Surface of protein–BPA Complex in MD simulations. (A) INS – BPA. (B) ESR1 – BPA. (C) SLC6A4 - BPA. (D) GRIA1 – BPA. (E) NTRK2 – BPA. All panels include: left, Gibbs energy landscape; middle, free energy landscape; right, electrostatic surface of the protein active site with the ligand shown as green sticks.

### 3.7. Validation using transcriptome data

To experimentally validate the clinical relevance of the five core genes, we analyzed four independent GEO datasets (GSE26063, GSE102556, GSE38206, GSE32280). Volcano plots (Fig 12A–F) showed significant differential expression in MDD patients compared with controls. GSEA of the GSE38206 dataset (Fig 13A–D) revealed that neurotrophic and insulin-related pathways were significantly downregulated in MDD patients. Importantly, the expression levels of INS, ESR1, SLC6A4, GRIA1, and NTRK2 were all significantly downregulated in MDD patients compared to healthy controls across multiple datasets (Fig 13E–J). These findings provide direct transcriptomic evidence linking the identified core targets to depression.

**Fig 12:**
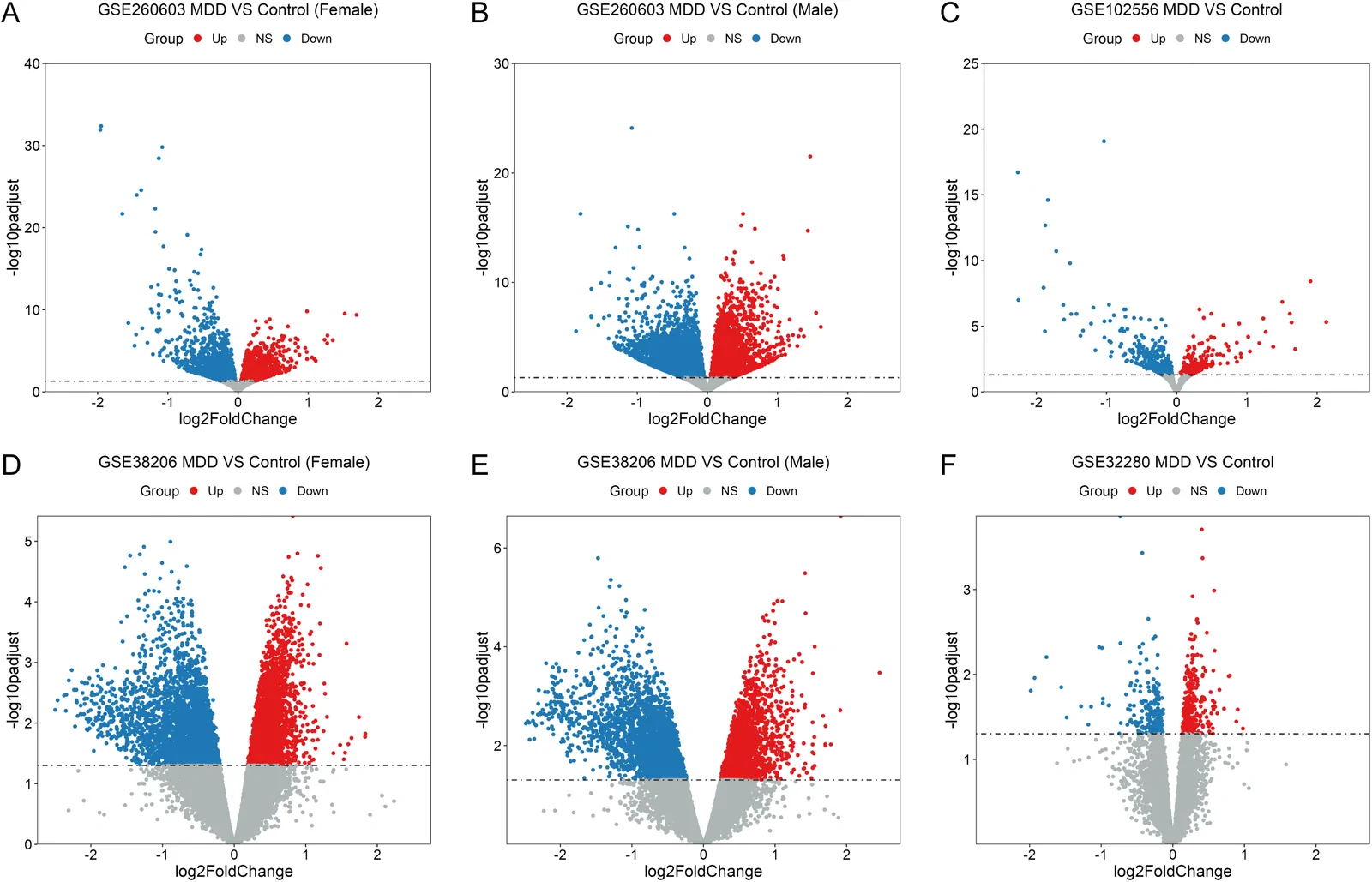
Volcano analysis of DEGs between MDD patients and healthy controls. (A-B): Volcano plots of differentially expressed genes in female and male MDD patients compared with normal controls in the GSE26063 dataset. (C): Volcano plot of differentially expressed genes in MDD patients compared with normal controls in the GSE102556 dataset. (D-E): Volcano plots of differentially expressed genes in female and male MDD patients compared with normal controls in the GSE38206 dataset. (F): Volcano plot of differentially expressed genes in MDD patients compared with normal controls in the GSE32280 dataset.

**Fig 13:**
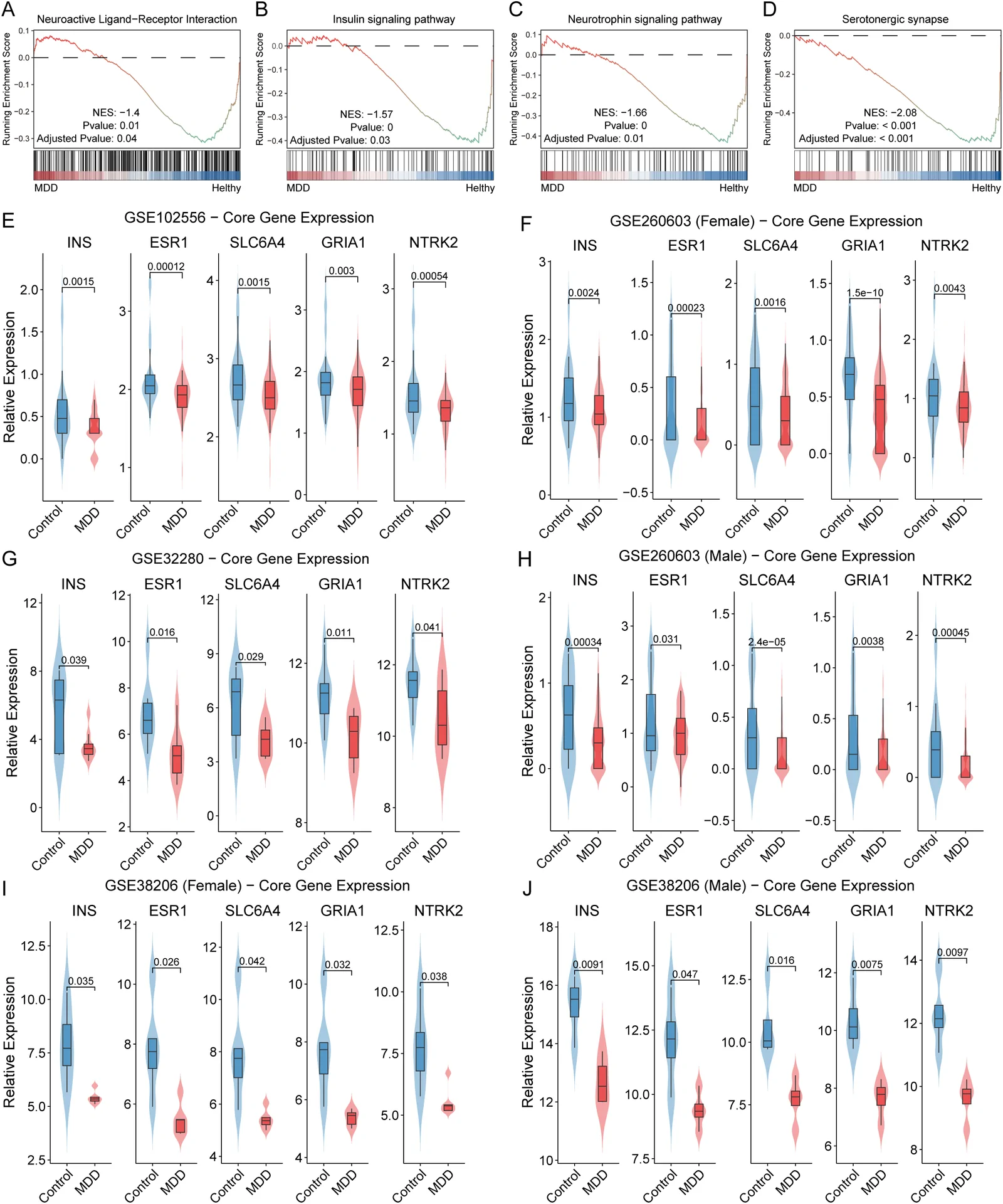
GSEA enrichment analysis and expression of core DEGs in MDD. (A–D): GSEA enrichment analysis of differentially expressed genes from the GSE38206 dataset showing that neurotrophic and insulin-related pathways are significantly downregulated in MDD patients. (E–J): The core genes INS, ESR1, SLC6A4, GRIA1, and NTRK2 are significantly downregulated in MDD patients.

### 3.8. In vivo experiments confirm that BPA downregulates five depression-related core molecules

The sucrose preference test and open field test confirmed that BPA exacerbates depressive-like symptoms, as evidenced by decreased sucrose intake, reduced total exploration distance in the open field, diminished exploration distance in the open area, and lower average exploration speed (Fig 14A). Western blot analysis demonstrated that BPA treatment at 50 mg/kg downregulated the protein expression of INS, ESR1, SLC6A4, NTRK2, and GRIA1 in mouse brain tissue (Fig 14B–C). qPCR further confirmed that BPA treatment at 50 mg/kg downregulated the mRNA expression of INS, ESR1, SLC6A4, NTRK2, and GRIA1 in mouse brain tissue.

**Fig 14:**
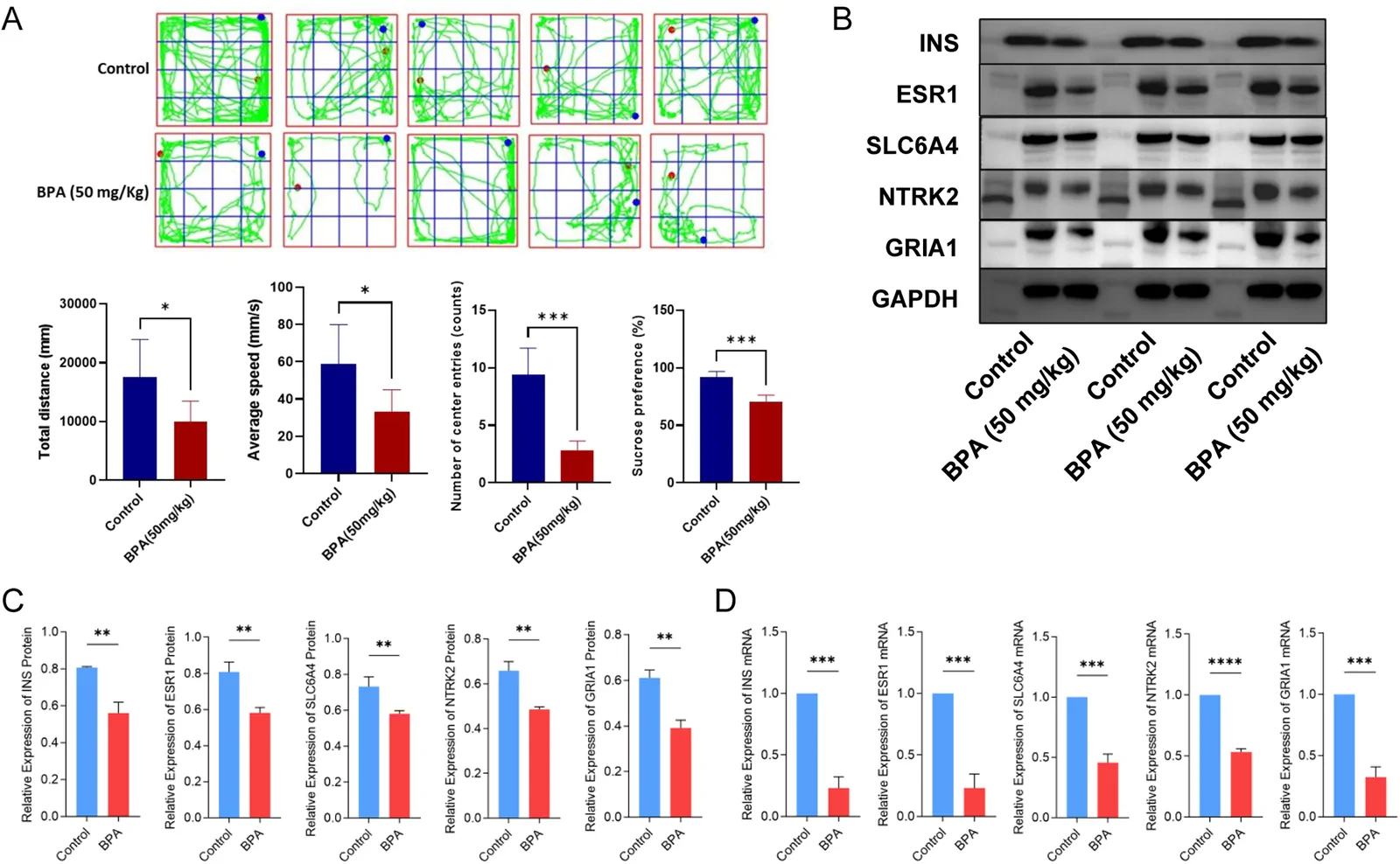
BPA aggravates depressive-like behaviors and downregulates core genes and proteins in mice. (A) The sucrose preference test and open field test confirmed that BPA exacerbates depressive-like behaviors, as evidenced by decreased total exploration distance in the open field, reduced average exploration speed, decreased exploration in the open area, and reduced sucrose intake. (B) Protein expression levels of the five core proteins (INS, ESR1, SLC6A4, NTRK2, and GRIA1) were significantly decreased after BPA intervention at 50 mg/kg. (C) mRNA expression levels of the five core genes (INS, ESR1, SLC6A4, NTRK2, and GRIA1) were significantly decreased after BPA intervention at 50 mg/kg.

## 4. Discussion

In this study, we integrated network toxicology, molecular docking, and molecular dynamics simulation to systematically investigate the molecular mechanisms underlying BPA-induced depression. Toxicity prediction confirmed BPA’s ability to cross the BBB and interact with estrogen receptors. By applying stringent target selection criteria (confidence ≥0.7), we identified 21 overlapping targets between BPA and depression, with five core hub genes—INS, ESR1, SLC6A4, GRIA1, and NTRK2—consistently emerging from PPI network analysis. Molecular docking of all 21 targets against BPA revealed that these five hub genes exhibited the strongest binding affinities (≤ –5.0 kcal/mol), and MD simulations with MM-GBSA calculations confirmed stable, energetically favorable binding. Importantly, independent GEO transcriptome datasets (GSE26063, GSE102556, GSE38206, GSE32280) demonstrated that all five core genes are significantly downregulated in MDD patients compared with healthy controls (Fig 12–13), providing direct clinical evidence linking these targets to depression. These findings not only identify key molecular targets of BPA neurotoxicity but also suggest that BPA may contribute to depression by interfering with insulin signaling, estrogen receptor function, serotonin transport, glutamatergic transmission, and neurotrophic support.

The selection of five hub targets from the 29 overlapping targets was based on the convergence of two independent criteria: PPI network degree centrality and BPA binding affinity. While several non-hub targets also showed favorable binding energies in blind docking (e.g., MYL3 at −8.2 kcal/mol, ESR2 at −7.8 kcal/mol), these were excluded for the following reasons. MYL3 encodes a cardiac myosin light chain whose expression is restricted to muscle tissue with negligible CNS expression [37], and it has no established role in depression pathogenesis. ESR2, although an estrogen receptor paralog with some reported associations with depression [38], is a lower-degree node in the PPI network and its mechanistic contribution to BPA-induced depression is less well-characterised in the literature compared with ESR1. The remaining non-hub targets similarly lacked either high PPI network centrality or established mechanistic links to depression. The five selected targets—INS, ESR1, SLC6A4, GRIA1, and NTRK2—represent the intersection of network topology and molecular binding evidence, providing the strongest rationale for further MD simulation and functional validation.

The insulin (INS) gene encodes a hormone critical for glucose homeostasis, but it is also synthesized in the CNS, where it exerts neuroprotective effects and modulates neuronal glucose uptake [39,40]. Insulin resistance is bidirectionally linked to depression: acute depressive episodes elevate insulin resistance, and depression exacerbates insulin resistance through inflammatory and HPA axis dysregulation [41–43]. Our molecular docking and MD simulations showed that BPA binds stably to INS with a binding energy of –5.8 kcal/mol and favorable MM-GBSA energy (–28.5 kcal/mol). This interaction may interfere with insulin’s conformation or receptor binding, potentially contributing to central insulin resistance. Our GEO validation further revealed that INS expression is significantly downregulated in MDD patients (Fig 13E–F). Given that exogenous insulin alleviates depressive-like behaviors in diabetic rats [44], BPA-induced INS downregulation or functional interference could represent a novel mechanism linking environmental BPA exposure to depression-associated metabolic dysregulation and diabetes-related central nervous system impairment [45].

Estrogen receptor alpha (ESR1) is a key mediator of estrogen signaling in the brain, particularly in the hypothalamus and amygdala, regions governing emotion and memory [46]. BPA is a well-known endocrine disruptor that binds to estrogen receptors, and our docking results confirmed strong binding to ESR1 (–7.2 kcal/mol), with MD simulations showing stable complex formation (RMSD ~0.2 nm, MM-GBSA –34.2 kcal/mol). BPA can act as a selective estrogen receptor modulator (SERM), and our findings suggest that BPA binding may alter ESR1’s transcriptional activity, leading to disrupted estrogen signaling. ESR1 polymorphisms are associated with depression susceptibility, severity, and treatment response, with notable sex and age specificity [47,48]. Female MDD patients exhibit altered hippocampal ESR1 mRNA levels [49]. Notably, our GEO analysis showed significant ESR1 downregulation in MDD patients (Fig 13E–F). BPA exposure has been linked to depressive-like behaviors in animal models, and our results provide a molecular rationale: BPA binding to ESR1 may interfere with estrogen-mediated serotonergic and noradrenergic modulation [50], thereby increasing vulnerability to depression, particularly in females. This aligns with the clinical observation that females are more sensitive to ESR1 variants [51].

The serotonin transporter gene SLC6A4, first mapped and characterized in early genetic studies [52,53], encodes the 5-HTT protein that regulates synaptic serotonin availability. The 5-HTTLPR short allele reduces gene transcription and increases depression risk [54]. Epigenetic methylation of SLC6A4 correlates with antidepressant efficacy and shows sex-specific patterns [55,56]. Our docking results demonstrated strong BPA binding to SLC6A4 (–6.3 kcal/mol), and MD simulations confirmed stable interaction over 100 ns with persistent hydrogen bonds. BPA-induced downregulation of SLC6A4 expression (validated in GEO datasets, Fig 13E–F) could reduce serotonin reuptake capacity, leading to synaptic serotonin depletion—a classic hallmark of depression. Interestingly, previous studies reported that BPA downregulates GABA(A)α2 receptor expression in the hippocampus and amygdala [22]. Together, these findings suggest that BPA may simultaneously impair both inhibitory (GABAergic) and serotonergic systems, creating an imbalance in mood-regulating circuits. The interaction between SLC6A4 and BDNF polymorphisms further influences depression risk [57]; our identification of NTRK2 (the BDNF receptor) as another BPA target raises the possibility that BPA disrupts BDNF–TrkB signaling in concert with serotonergic dysfunction.

Glutamate receptor GRIA1 encodes the GluA1 subunit of AMPA receptors, which mediate fast excitatory transmission and synaptic plasticity [58]. Enhanced AMPA receptor activation and GluA1 expression are associated with ketamine’s rapid antidepressant effects [59], whereas increased GRIA1 expression in the dorsolateral prefrontal cortex of MDD patients suggests region-specific dysregulation [60]. Chronic pain-induced depression involves elevated GRIA1 and phosphorylated GRIA1 in the amygdala, driving pathological excitatory plasticity [61]. Our docking and MD simulations showed that BPA binds GRIA1 with high affinity (–6.0 kcal/mol) and remains stably associated with the ligand-binding domain. GEO data confirmed that GRIA1 is significantly downregulated in MDD patients (Fig 13E–F). BPA-induced downregulation of GRIA1 could impair AMPA receptor function, disrupting excitatory/inhibitory balance in prefrontal-limbic circuits [62]. Given that BPA also downregulates GABA(A)α2 receptors [22], the combined effect on glutamatergic (GRIA1) and GABAergic systems may tip the network toward hyperexcitability or reduced plasticity, both implicated in depression pathophysiology.

Neurotrophic receptor tyrosine kinase 2 (NTRK2) encodes TrkB, the high-affinity receptor for BDNF, which is essential for neuroplasticity and stress resilience [63]. NTRK2 polymorphisms (e.g., rs1565445, rs1948308) are associated with treatment-resistant depression and reduced hippocampal volume [64,65]. The BDNF–NTRK2–CREB1 pathway is critical for antidepressant responses, and genetic variants increase rumination and depression risk [66]. Our docking results showed strong BPA binding to NTRK2 (–5.5 kcal/mol), and MD simulations revealed stable complexation with favorable free energy. GEO validation demonstrated NTRK2 downregulation in MDD patients (Fig 13E–F). BPA-induced interference with TrkB function could impair BDNF signaling, reducing neurogenesis and synaptic plasticity in the hippocampus and prefrontal cortex—core neurobiological deficits in depression. Notably, BPA’s downregulation of androgen receptor and GABA(A)α2 receptors [22] may synergize with TrkB dysfunction, as these pathways converge on synaptic stability and mood regulation.

Beyond individual targets, our GO and KEGG enrichment analyses revealed that the overlapping genes are significantly enriched in neural synaptic function, neurotransmitter binding, and the neuroactive ligand-receptor interaction pathway—all of which are central to depression pathogenesis [62,67–69]. Synaptic homeostasis, a key negative feedback mechanism that adjusts synaptic strength to counteract excessive excitation or inhibition, is disrupted in depression [68]. BPA’s ability to simultaneously bind and potentially downregulate INS, ESR1, SLC6A4, GRIA1, and NTRK2 suggests a multi-hit mechanism: BPA may impair insulin-mediated neuroprotection, disrupt estrogen-dependent monoamine modulation, reduce serotonin reuptake capacity, alter glutamate receptor function, and blunt BDNF–TrkB neuroplasticity. The convergence of these effects on mood-regulating circuits (hippocampus, amygdala, prefrontal cortex) provides a plausible explanation for the epidemiological association between BPA exposure and depression [14,20,21].

Several limitations of this study should be acknowledged. First, our findings are based partly on computational predictions and in silico simulations and therefore require further mechanistic validation. Second, one 100 ns production MD trajectory was analyzed for each protein–BPA complex; independent replicate trajectories were not performed. Accordingly, the MD results should be considered exploratory computational evidence, and future studies should include independent replicate simulations to more rigorously assess reproducibility. Third, ligand-displacement analysis demonstrated target-dependent stability and showed substantial movement of BPA from the initial docked pose in the INS complex, emphasizing that favorable docking or MM-GBSA energies should not be interpreted as independent proof of persistent binding. Fourth, the GEO datasets confirmed differential expression of the five core genes in MDD patients, but these data do not directly demonstrate causality between BPA exposure and target downregulation. Future studies should examine whether BPA exposure correlates with reduced expression of these genes in human populations and whether restoring their function can ameliorate BPA-induced depressive-like phenotypes. Finally, BPA often coexists with other EDCs (e.g., phthalates); investigating their synergistic or additive effects on depression pathogenesis will be an important next step. Despite these limitations, our study provides an exploratory computational and experimental framework and identifies specific, testable molecular targets for understanding BPA neurotoxicity in depression.

## 5. Conclusions

Our study integrated network toxicology, molecular docking and molecular dynamics simulation techniques to investigate the potential molecular mechanisms underlying the association between bisphenol A (BPA) exposure and the pathogenesis of depression. A total of 29 targets mediating the effects of BPA on depressive pathogenesis were identified, among which the key hub targets included INS, ESR1, SLC6A4, GRIA1 and NTRK2, and the core pathways comprised neural synaptic pathways, neurotransmitter binding pathways and the neuroactive ligand-receptor interaction pathway. These findings provide novel insights into the role of BPA in the pathogenesis of depression and lay a theoretical foundation for the drug development and clinical treatment of depression. However, further experimental validation using basic and clinical in vitro and in vivo models is required to verify these observations.

## Supporting information

S1 Fig AutoDock vina blind docking: BPA vs. All 29 overlapping targets. (PNG)

S2 Fig BPA internal conformation RMSD (ESR1,GRIA1,NTRK2,SLC6A4;INS data unavailable) (A) Ligand internal RMSD of BPA heavy atoms relative to the initial docked pose for ESR1, GRIA1, NTRK2, and SLC6A4 over 100 ns MD simulations (INS ligand RMSD data unavailable). (B) BPA–protein RMSD showing displacement of BPA from the initial docked pose for all five hub targets over 100 ns. Dashed line indicates the 0.3 nm reference threshold.
(PNG)

S1 File Supplementary data tables. This document provides a detailed list of the toxicity mechanisms of BPA predicted by the ProTox platform.
(PDF)

S2 File Supplementary data tables. This document presents a comprehensive list of the BPA-induced toxicity mechanisms predicted via the ADMETlab platform.
(PDF)

S3 File Supplementary data tables. This Excel file includes the following data sheets: 1) BPA-related targets, 2) Depression-related disease targets, and 3) Overlapping targets between BPA and depression.
(XLSX)

S4 File Supplementary data tables. This Excel file includes the following data sheets: Molecular docking binding energies of BPA against all 29 overlapping targets.
(XLSX)

S5 File Supplementary data tables. This Excel file includes the following data sheets: AutoDock Vina grid box parameters (center coordinates and dimensions) for focused molecular docking of BPA against the five hub targets and lysozyme docking control.
(XLSX)

S6 File Supplementary data tables. This Excel file includes the following data sheets: Positive Control and Docking Control.
(XLSX)

S7 File Supplementary data tables. This Excel file includes the following data sheets: RMSF peak regions mapped to functional domains and binding pocket residues for the five hub protein–BPA complexes.
(XLSX)

S8 File Supplementary data tables. This Excel file includes the following data sheets: MM-GBSA binding free-energy decomposition for all five hub protein–BPA complexes from 100 ns MD simulations, including per-frame data and mean ± SD values.
(XLSX)

S9 File Supplementary data tables. This Excel file includes the primer sequences used for quantitative real-time PCR (qPCR) validation of the five hub target genes.
(XLSX)
